# Effect of Thermal Treatments and Ion Substitution on Sintering and Crystallization of Bioactive Glasses: A Review

**DOI:** 10.3390/ma16134651

**Published:** 2023-06-28

**Authors:** Francesco Gerardo Mecca, Devis Bellucci, Valeria Cannillo

**Affiliations:** Dipartimento di Ingegneria Enzo Ferrari, Università degli Studi di Modena e Reggio Emilia, Via P. Vivarelli 10, 41125 Modena, Italy; francescogerardo.mecca@unimore.it

**Keywords:** bioactive glasses (BGs), thermal treatments, sintering, crystallization, ion substitution

## Abstract

Bioactive glasses (BGs) are promising materials for bone regeneration due to their ability to bond with living bone tissue. However, thermal stability and mechanical properties of BGs need improvement for better clinical performance. In this paper, we present an overview of the influence of different ions on the sintering and crystallization of BGs. Specifically, this review focuses on the impact of thermal treatments on the crystallization of 45S5 and other significant BG compositions. Potential applications of these thermally treated BGs, such as scaffolds, BG-based composites, and thermally sprayed coatings, are explored. Moreover, the substitution of ions has been investigated as a method to enhance the thermal properties of BGs. Notably, zinc, potassium, and strontium have been studied extensively and have demonstrated promising effects on both the thermal and the mechanical properties of BGs. However, it is important to note that research on ion inclusion in BGs is still in its early stages, and further investigation is necessary to fully comprehend the effects of different ions on sintering and crystallization. Therefore, future studies should focus on optimizing the ion substitution method to improve the thermal, mechanical, and even biological properties of BGs, thereby enhancing their potential for various biomedical applications.

## 1. Introduction

“If you can make a material that will survive exposure to high-energy radiation, can you make a material that will survive exposure to the human body?”. This question, asked by U.S. Army Medical Corps Colonel Klinker, led to a ground-breaking idea taking shape in Dr. Larry L. Hench’s mind: the first bioactive glass (BG), synthesized in 1969, and today worldwide known as Bioglass^®^ or 45S5 [1]. The composition of 45S5, expressed in mol.%, is as follows: 46.1 SiO_2_, 26.9 CaO, 24.4 Na_2_O, and 2.6 P_2_O_5_. Even today, 45S5 continues to serve as a reference point for newly developed bioactive glasses (BGs). This composition was attained thanks to a 2 years research effort, through the analysis of the Na_2_O–CaO–SiO_2_ system, where 45S5 sits near a ternary eutectic [1,2]. Phosphorus was originally included by Hench in the glass composition, among other reasons, due to its vital role as a constituent of hydroxyapatite (HA), the mineral phase of bone tissue [1].

The discovery of bioactive and healing properties of 45S5 revolutionized the world of biocompatible materials. Prior to this discovery, it was widely believed that the only materials suitable for use inside the human body were those that did not react with it. Consequently, past research efforts primarily focused on identifying the most nonreactive materials, mainly amongst metals and plastics, such as titanium and ultrahigh-molecular-weight polyethylene (UHMWPE) [3]. These materials are now categorized as first-generation biocompatible materials. In contrast, second-generation biocompatible materials, such as bioactive glasses, have the ability to induce a controlled action–reaction when in contact with living tissue. Some BGs can even be engineered to become third-generation biocompatible materials. The key difference between second- and third-generation BGs lies in the latter’s ability to activate genes and promote cell proliferation, while stimulating the regeneration of living tissues [4]. Thus, BGs used in tissue engineering (TE) can exhibit essential properties such as osseointegration, osteoconductivity, and osteoinductivity. These terms describe, respectively, the ability of a material to bond with bone without the formation of fibrous tissue, the surface’s capacity to facilitate bone growth, and the capability to stimulate stem cells to differentiate into bone-forming cells [5]. BGs have been mainly studied for their bioactivity. A material is considered bioactive when it has the ability to elicit a specific response from living tissue. In particular, BGs are considered bioactive for their ability to bond to bone tissue, forming bone-like apatite when grafted into a body [6]. Kokubo et al. introduced a straightforward in vitro test method for the initial evaluation of a material’s bonding capacity with bone. This method assesses the material’s ability to form apatite by exposing it to a solution called simulated body fluid (SBF), which consists of salts dissolved in water [7]. Although some materials can bond without apatite formation, the test remains a reliable indicator of bioactivity, making it a valuable tool for evaluating the bioactivity of BGs [8].

Third-generation bioactive glasses, including mesoporous bioactive glasses (MBGs), have shown remarkable potential in stimulating angiogenesis, as evidenced by in vitro and in vivo testing [9]. Furthermore, MBGs can be engineered for drug delivery when in contact with living tissue, thanks to their nanoporous structure. For example, Hu et al. designed a selenium MBG where tetravalent selenium can improve bioactivity while having a selective cytotoxicity to cancer cells [10]. Additionally, some BGs, such as S53P4, a commercially available glass marketed as BonAlive^®^, exhibit antibacterial properties. With a composition (mol.%) of 53.8 SiO_2_, 21.8 CaO, 22.7 Na_2_O, and 1.7 P_2_O_5_, S53P4 demonstrates high bioactivity and a significant release of ions. This, in turn, raises the pH level in the surrounding area, leading to an effective antibacterial effect observed in in vitro testing [11]. These findings underscore the considerable untapped potential of this class of materials.

BG powders are frequently subjected to thermal treatments, typically involving exposure to temperatures above 600 °C [12]. These treatments are essential to facilitate the sintering process, which leads to the formation of a dense three-dimensional (3D) structure [13]. This is required in different applications, such as scaffold manufacturing, thermal spraying of BG powders and manufacturing of glass–ceramic composites. 

The behavior of 45S5 bioactive glass powder during thermal treatments has been thoroughly studied. Crystallization is a detrimental phenomenon that occurs when BG powders are heated beyond their crystallization onset temperature (i.e., the temperature at which the crystallization peak starts, T_c,onset_), and it is most prominent at the crystallization peak temperature (T_c_) [14,15]. In fact, crystallization interferes with the bioactivity and mechanical performance of sintered BG powders, as well as impedes viscous flow (i.e., if crystallization occurs, sintering may be slower). Since sintering and crystallization are closely related, they are also referred to as a single process, namely, sinter-crystallization [16]. Several studies focus on the “sintering window” (i.e., the difference between the crystallization temperature T_c,onset_ and the glass transition temperature T_g_) to address the issue of obtaining sintered artefacts with limited or no crystallization happening in the glass structure. 

Various ions have been extensively investigated for their ability to widen the sintering window and effectively modify the composition of the glass. The “network connectivity” (NC) model has proven valuable in elucidating the impact of different ions on the structural characteristics of the glass. NC describes the average number of oxygen atoms, acting as a bridge in the network. NC is equal to 4 for pure silica [17]. Hill and Brauer [18] developed this model based on experimental data by Watts et al. [19], condensed in the following equation [19,20,21]:

(1) $$\mathrm{NC}=\frac{\left[4\cdot \left({\mathrm{Si}O}_{2}\right)+6\cdot \left(P_{2}O_{5}\right)\right]-2\cdot [M_{2}O+M’O]}{\mathrm{Si}O_{2}},$$

where “SiO_2_” and “P_2_O_5_” represent the molar percentages of the respective oxides present in the glass composition, and “M_2_O” and “M’O” denote the alkali and alkali-earth metal oxides, respectively. The NC of bioactive glasses typically ranges from 2 to 3. A higher value indicates higher connectivity and, thus, better thermal properties, but worse bioactivity. A lower value indicates reduced connectivity, leading to lower thermal properties; however, this also enhances reactivity and bioactivity by increasing the solubility of the glass and facilitating ion release from the glass [22]. 

This paper aims to examine the influence of thermal treatments on BGs, with a specific focus on their effect on the glass structure, sintering, and crystallization. Furthermore, this review investigates the modifications made to the composition of BGs by incorporating specific ions into the glass structure and examines the consequent effects on sintering and crystallization processes. Additionally, the review briefly analyzes potential applications that involve subjecting BGs to high temperatures, such as scaffold manufacturing, thermally sprayed coatings, and BG/ceramic composites manufacturing. Due to the high temperatures involved, the feasibility of these applications is closely associated with the thermal properties of the BG, making it crucial to examine their effects in detail.

**Figure 1:**
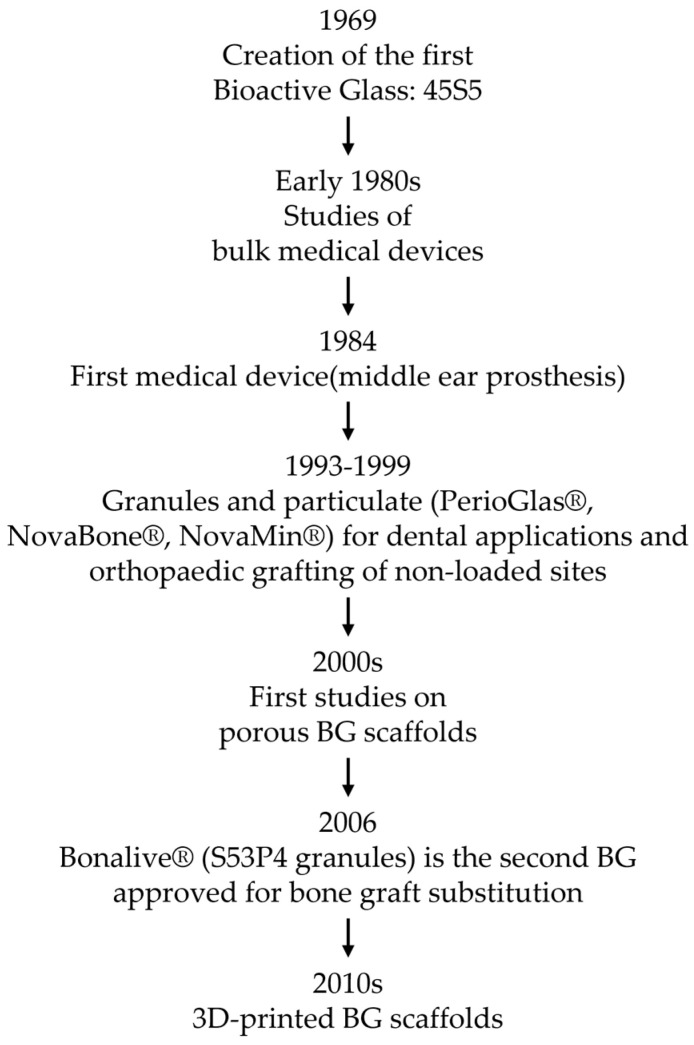
Flowchart of the timeline of the major breakthroughs in the bioactive glass field [22].

For a better understanding of the significant progress achieved in the field of BGs, Figure 1 presents a flowchart showing the major breakthroughs and significant milestones attained throughout the evolution of bioactive glass.

## 2. Why Thermal Treatments?

As previously mentioned, thermal processing is a necessary step for shaping bioactive glass powder into the desired form, depending on the application. Furthermore, the thermal treatment of BG powder triggers the formation of a liquid phase. Upon solidification, this liquid phase facilitates the sintering of the powder, thereby improving the mechanical properties of the BG material. [23]. Many research papers have addressed the problem of thermally treating BG powders while minimizing the negative effects of crystallization, which can significantly impair the BG’s final properties. Thus, striking the right balance between promoting sintering and preserving the mechanical properties and bioactivity of BG necessitates meticulous control over thermal treatment processes, along with extensive research into different BG compositions. This equilibrium is of utmost importance in the development of bioactive glasses with optimal properties tailored for diverse biomedical applications.

Scaffolds are biodegradable porous 3D structures that can be made from various materials, including bioactive glass powders. Scaffolds serve as templates in large bone defects, to induce cell proliferation and vascularization of the damaged tissue [24]. Thus, using scaffolds may help replicate cancellous bone structure and permit cell proliferation [25,26,27]. The development of an optimal scaffold for bone repair is a complex process that involves careful control of the properties of the materials and parameters used in their manufacturing. BG powders have shown great potential as a material for bioactive scaffold manufacturing due to their ability to enhance bone tissue regeneration. However, achieving the desired properties requires careful thermal treatment and compositional control. The desired properties of an ideal scaffold have been extensively discussed in the literature [28,29]. Certain critical properties include the capacity to host cells, biocompatibility, and bioresorbability (i.e., the ability to dissolve in a biological environment without leaving behind harmful byproducts). Another crucial aspect is the high porosity of the scaffold, ideally surpassing 90%, coupled with a minimum pore size of 100 µm, to facilitate cell infiltration. Moreover, the scaffold must possess mechanical properties similar to that of bone [5]. 

Heat treatments are essential in the majority of manufacturing technologies for producing BG scaffolds for bone tissue engineering. The main techniques used today to produce scaffolds are foam replica, additive manufacturing, sol–gel, freeze-casting, and electrospinning [30]. Among these protocols, the foam replica and additive manufacturing techniques are the most frequently used ones to manufacture BG scaffolds. The foam replica technique and several additive manufacturing techniques produce a product known as a “green body”. However, to achieve the final scaffold form with proper consolidation and improved mechanical properties, the raw “green body” must undergo a crucial heat treatment process [22,30,31,32,33].

Bioactive glass powders have also been used to coat bioinert metallic implants. This application has been studied to improve the mechanical performance of inorganic metallic grafts. Usually, the metallic materials used to manufacture these grafts (alloplastic implants), such as titanium and magnesium, promote the formation of fibrous tissue [12,34]. Coating a metallic graft with a BG film may improve the chemical stability of the metallic substrate, providing a physical barrier to avoid in-body corrosion. This protective coating also reduces the risk of prosthesis rejection. The bioactivity of the BG can also improve the adhesion of the implant, as the BG coating is resorbed, and a superficial HA layer can form. Thus, the coating may help improving the general stability of the implant and attaining biological fixation [35]. 

Various techniques are utilized to deposit a surface coating of BG powder onto a metallic substrate. These techniques include enameling, sol–gel, electrophoretic deposition, plasma spraying (PS), and laser cladding. Enameling, PS, and laser cladding require exposing the powders to heat in order to sinter or melt the glass and create the superficial layer [12,36]. Enameling is a widely recognized and traditional technique that involves the deposition on a substrate of a coating BG-containing slurry, which is subsequently dried and sintered. This process typically employs temperatures in the 800–900 °C range [37]. PS and thermal spraying techniques are both reliable methods for depositing BG coatings onto metallic inert substrates, in order to manufacture composite load-bearing grafts. The metallic substrate provides mechanical strength, while the bioactivity of BG ensures bone-bonding ability [12]. PS is an advanced technique that involves applying high voltages on inert gases to create a plasma, which can reach extremely high temperatures (thousands of degrees Celsius). This intense heat melts the powders while rapidly accelerating the particles toward the surface, where the melt droplets quickly solidify. Nevertheless, the BG remains at high temperatures for an extended duration, which can result to the partial crystallization of the glass [38,39]. 

Laser cladding, a more recent technique, operates at lower temperatures, typically ranging from 1000 to 1500 °C. A critical aspect of laser cladding technology is the risk of post-treatment cracking of the coating [40]. This occurs due to the significant difference in the coefficient of thermal expansion (CTE) between the BG and the substrate material. This may cause delamination of the coating layer. Solutions to mitigate this issue include (i) modifying the glass’ composition to match its CTE with that of the substrate, (ii) depositing a “bond coat” with an intermediate CTE, (iii) implementing mechanical modifications to the substrate’s surface, through roughening or patterning, and (iv) using chemical alterations to the substrate’s surface [36]. Furthermore, Foppiano et al. successfully deposited a functionally graded BG coating, where silica content decreased while the coating thickness increased [41]. Currently, commercially available solutions for bioactive glass coatings on metallic implants are limited. Ongoing efforts are being made to improve the techniques and materials in order to optimize the results [12].

Over the past three decades, researchers have explored the potential of BG as a secondary phase in glass–ceramic composites, in combination with hydroxyapatite (HA) or tricalcium phosphate (β-TCP) as the primary phase [42,43]. Hydroxyapatite (Ca_10_(PO_4_)_6_(OH)_2_) is a calcium-based mineral, chemically similar to biological apatite, which is the main mineral phase in bone tissue. Tricalcium phosphate (Ca_3_(PO_4_)_2_) is a salt that can interact with living tissue and be resorbed. Both HA and TCP have been extensively studied and utilized in clinical settings due to their ability to promote bone growth in in vivo studies. This ability, commonly referred to as osteoconductivity, has made them candidates for the use in bone tissue engineering [44,45]. Interestingly, HA and TCP exhibit distinct performance in terms of reactivity. For instance, HA is more stable in contact with living tissue, displaying lower dissolution rates. Conversely, TCP is more reactive, exhibiting rapid dissolution rates in contact with physiological pH [46]. This disparity in reactivity can be exploited to manufacture TCP/HA ceramic composites. In fact, by selecting specific TCP/HA ratios, it becomes possible to carefully tailor the composite’s properties to meet specific requirements [47]. 

Bioactive glass–ceramic composites offer promising potential for bone tissue engineering. The primary motivation for using bioactive glass–ceramic composites is to enhance the mechanical properties of individual phases while concurrently tailoring the bioactivity of the composite by adjusting the content fraction of each phase. Studies indicate that an optimal fraction of ceramic particles, approximately 40 vol.%, results in the highest strength for the composite [48]. The inclusion of BG in the composite can offer advantages as it can improve the densification of the ceramic phase, acting as an effective sintering aid, which in turn leads to a final product with improved mechanical properties [49,50]. For instance, the incorporation of as little as 2 wt.% bioactive glass into ceramic powders can substantially enhance the flexural bend strength of the composite [51]. 

The fraction of BG in the composite has a direct impact on the sintering rate of HA/BG composite. In fact, the presence of BG can influence the grain size of HA, leading to smaller grains, thereby enhancing the strength and sintering quality of the final material [51]. Furthermore, Baino et al. evinced that the presence of BG in TCP/BG composites may help improve the stability of β-TCP phase up to 1300 °C [52]. Without the stabilizing effect of the bioactive glass, the β phase would irreversibly convert to α phase around 1150 °C [52]. 

Additionally, the distinctive properties of BG, such as its ability to induce bone formation (in other words, the aforementioned osteoinductivity) and the presence of specific ions in the glass’s composition, can be leveraged to further optimize the composite’s performance [53,54]. Thus, ongoing research on BG properties and applications has great potential for advancing biomaterials and regenerative medicine.

## 3. Effects of Crystallization of BGs

As we mentioned in Section 1, crystallization is considered to have a detrimental effect on bioactive glasses on several levels. Even though the crystallization peak temperature is higher than the glass transition temperature, in many cases, the crystallization of BGs takes place before significant densification can be achieved through sintering [23]. The main negative effects are (i) a modest change in volume (typically, the crystalline phase exhibits a distinct density in comparison to its amorphous counterpart), and (ii) loss of mechanical integrity in porous scaffolds, caused also by their brittleness [55]. Thus, the process of crystallization can have adverse effects, especially on the mechanical integrity of sintered products; for instance, the volume variation may create internal stresses, which, combined with the brittleness, severely hinder the mechanical performance of the samples.

From a strictly biological perspective, crystallization does not inhibit the bioactivity of bioactive glasses, but rather slows it down. This delay in bioactivity also affects the conversion of the glass’s outermost layer into hydroxyapatite once the material is implanted in the body and comes into contact with biological fluids. Peitl et al. found that crystallization slowed down the deposition onset of HA by three to four times in BGs containing phosphorus [56]. The slowing down of HA deposition onset is problematic since it could result in nonuniform dissolution of the scaffold, which, combined with the loss of mechanical performance, makes it unsuitable for human graft implantation. All the abovementioned effects are nevertheless linked to different parameters, such as heat treatment temperature and time; in fact, increasing the treatment time results in higher levels of densification, as well as higher crystallite and grain size. These effects do not appear to follow a linear trend over sintering time. In a study by Hashmi et al., it was observed that the crystallization rate of the BG (at a dwell temperature of 1000 °C) is higher in the 5–10 h range compared to the 0–5 or 10–15 h ranges [57]. However, as demonstrated by the wide range of applications mentioned above, the use of high temperatures is crucial in establishing the necessary sintering conditions for the manufacturing of BG delivery solutions. 

One critical factor to consider is the narrow temperature range between the glass transition temperature (T_g_) and the crystallization temperature (T_c,onset_), i.e., the aforementioned sintering window. In fact, this process parameter plays a crucial role in achieving successful sintering of the glass. In this regard, commercial bioactive glasses such as 45S5 and S53P4 have extremely narrow sintering windows, which makes it difficult to subject these materials to thermal treatment without inducing crystallization. In order to expand the sintering window, researchers have directed their efforts toward developing new BG compositions or modifying the existing ones. The objective is to achieve BGs with wider sintering windows, thus allowing for successful sintering while limiting the crystallization process [58].

### 3.1. Crystallization of 45S5

Over the last few years, extensive research has been conducted on 45S5, with particular emphasis on its response to heat treatment. Previous studies have identified two distinct crystallization mechanisms for BGs, namely bulk and surface crystallization [59]. While the factors influencing the dominance of one mechanism over the other are not yet fully understood, it is evident that variations in granulometry and composition can have a significant influence on their respective prevalence. Different research papers highlighted a significant difference between the heat treatment of bulk and powdered 45S5 [23,55,59,60]. This disparity is attributed to the size and granulometry of the sample, which play a crucial role in determining the outcome of the treatment. For instance, smaller granulometries (<300 µm) of 45S5 tend to undergo surface crystallization, whereas bulkier samples display bulk crystallization [55]. This disparity is evident when analyzing the differential thermal analysis (DTA) data for coarse and fine BG powders. In fact, Massera et al. reported a shift of the crystallization peak toward lower temperatures, coupled with a widening of the peak of 45S5 and S53P4 [61]. However, it should be noted that the crystallization mechanism of 45S5 is generally complex, involving a combination of different crystallization mechanisms [55].

Through analyzing DTA, differential scanning calorimetry (DSC), and X-ray diffraction (XRD) pattern data, it is possible to evaluate the characteristic temperatures and crystalline phases of a given bioactive glass. In past years, it was debated what the major crystalline phase was. While several authors agreed about identifying combeite (Na_2_Ca_2_Si_3_O_9_) as the main crystalline phase [56,62,63], more recent research by Li et al. [64] pointed out that, for lower temperatures in the 600–700 °C range, the main crystalline phase is actually Na_2_CaSi_2_O_6_ [23,64]. Today, it is widely accepted that, during the heating of 45S5 bioactive glass, two distinct crystallization processes occur. Silicorhenanite (Na_2_Ca_4_(PO_4_)_2_SiO_4_) has been identified as the second phase, with a nucleation temperature of about 850 °C [23,62]. The previous misinterpretation of combeite as the main crystalline phase may be attributed to the structural similarity between Na_2_Ca_2_Si_3_O_9_ and Na_2_CaSi_2_O_6_, leading to ambiguous XRD patterns [62,65].

Powdered 45S5 tends to have a slightly different behavior, with respect to bulkier forms (such as dense glass, granules > 300 µm, or fritted glass). This can be attributed to the presence of small-sized particles, which enable additional thermal phenomena, including sintering, to take place. As previously mentioned, sintering is a process of densification performed on powders, at temperatures below their melting point. It aims to consolidate the powders, leading to a more compact and durable material. There are three conditions that must be met for powder sintering [66]: (i) the presence of a liquid phase that coexists with a solid phase. This occurs when the powder particles begin to melt on the surface; (ii) a decrease in the free enthalpy of the system, as this process becomes spontaneous at sufficiently high temperatures; (iii) the structural properties of the sintered product must be comparable to those of a compact material.

Without going into the details, the sintering behavior of a specific material can be evaluated through heating microscopy, which involves measuring the shrinkage of a compacted powder cylinder using the following equation [67]:

(2) $$S_{T}=\frac{\Delta A}{A_{0}}\;\times \;100,$$

where S_T_ is the total shrinkage, and ΔA is the difference between the area of the sample at room temperature (A_0_) and the area of the sample at high temperature (A) [67]. Among the main studies dedicated to the crystallization of 45S5, we can mention Lefebvre et al. [15,62]. In their research, the authors analyzed the five distinct stages observed in the crystallization process of this bioactive glass, utilizing DTA data. These stages include the initial glass transition occurring at 550 °C, a glass-in-glass phase separation at 580 °C, two crystallization phases at 610 °C and 800 °C, a secondary glass transition observed at 850 °C, and melting at 1200 °C [62]. According to the available data, the sintering window of 45S5 is very narrow, covering a temperature range of only 60 °C. Consequently, it may not be the most suitable option for fabricating sintered bioactive glass manufacts. Furthermore, we would like to stress that the powder’s chemistry, shape, and granulometry deeply affect the sintering and crystallization kinetics [68,69]. This fact is of crucial relevance for the optimization of more complex structures, such as scaffolds that usually require a sintering process to consolidate.

### 3.2. Increasing the Thermal Stability of 45S5: The “Sol–Gel” Option?

To enhance the thermal stability of 45S5, researchers have undertaken investigations into the sol–gel method as a viable alternative to conventional melting technology. The sol–gel process involves the chemical synthesis of the glass using liquid and powdered precursors. This approach offers great flexibility in modifying the composition of the final glass by simply adjusting the precursor materials and incorporating various ions into the glass structure. However, it is important to note that the sol–gel process also requires a thermal treatment to trigger the chemical decomposition of the precursor reagents. In the case of 45S5, this thermal treatment may be conducted at a temperature lower than the glass’s crystallization temperature [70]. Consequently, the end result of this process is a fully amorphous glass rather than a partially crystallized one.

In addition to crystallization, the elevated temperatures reached during the treatment can induce various phenomena in the bioactive glass. These include partial crystallization, sintering, shrinkage, and the evaporation of humidity and CO_2_, resulting in significant mass loss [70,71]. For example, Cacciotti et al. successfully synthesized 45S5 using the sol–gel method and reported a shift of the crystallization onset point to over 800 °C [70]. These results were confirmed in subsequent research by Lombardi et al., where sol–gel-derived 45S5 showed crystallization of Na_2_Ca_2_Si_3_O_9_, with traces of Na_2_Ca_4_(PO_4_)_2_SiO_4_ and SiO_2_ after heat treatment at 900 °C [71]. Conversely to previous results, more recent research by Nawaz et al. reported the formation of combeite (Na_2_Ca_2_Si_3_O_9_) and rhenanite (NaCaPO_4_) at 600 °C, and combeite and Na_2_Ca_2_Si_2_O_7_ at 700 °C [72]. This fact can be probably ascribed to the use of different treatment times or granulometry which is known to heavily influence the behavior of the glass.

## 4. The Effect of Thermal Treatments on Some Relevant Compositions

In the last 20 years, researchers focused on developing new BGs to achieve a widening of the sintering window, which can enhance the properties and processability of BGs [22]. The properties of S53P4, a commercially available bioactive glass with a composition (in mol.%) of 53.8 SiO_2_, 21.8 CaO, 22.7 Na_2_O, and 1.7 P_2_O_5_, have been studied for almost three decades [73]. Lindfors et al. also highlighted its antibacterial properties in bone tissue infection therapy [11]. Massera et al. conducted extended analysis of the behavior of S53P4 bioactive glass, comparing it to the “gold” standard 45S5 [61]. The authors found that the behavior of S53P4 significantly deviates from that of 45S5. Although the sintering window of the S53P4 glass is wider than that of 45S5, it is not wide enough to achieve sintering of the BG while preserving its amorphous nature.

To explore the sintering behavior of S53P4, researchers conducted a series of experiments and captured multiple micrographs of the powders at different sintering stages. These micrographs provided valuable insights into the progression of sintering, showcasing the distinct stages of sintering and the formation of necks between the particles [74]. After the heat treatment, the primary crystalline phase observed was Na_2_Ca_2_Si_3_O_9_. S53P4 exhibited surface crystallization of needle-like combeite crystals. This crystallization process occurred through a relatively straightforward nucleation and growth mechanism, in contrast to 45S5, where crystallization mechanisms tend to be more intricate. This finding implies that the properties of S53P4 may be less influenced by variations in grain size compared to other bioactive glasses, ultimately impacting the material’s overall performance. Nevertheless, it is important to highlight that reducing the grain size still resulted in a lower crystallization temperature [61].

Brink and colleagues developed a novel bioactive glass, with the aim of improving the thermal properties of 45S5, called 13-93 (composition in wt.%: 53.0 SiO_2_, 20.0 CaO, 6.0 Na_2_O, 4.0 P_2_O_5_, 12.0 K_2_O, and 5.0 MgO). Today, this composition is approved for in vivo use in Europe [75,76]. Fu et al. conducted DTA on the 13-93 bioactive glass, revealing a glass transition temperature (T_g_) of approximately 606 °C and a crystallization onset temperature (T_c,onset_) around 714 °C. These findings indicate a sintering window of approximately 108 °C [77]. The prevailing opinion suggests that 13-93 exhibits a significantly lower propensity for crystallization when compared to 45S5. However, there is a lack of consensus regarding the presence of a well-defined sintering window for this particular bioactive glass. This discrepancy arises from the broad shape of the crystallization peak observed during DTA [58].

Fagerlund et al. [78] conducted a more in-depth analysis of 13-93 BG, comparing its performance to another newly developed BG, named 1-98 (composition in wt.%: 53.0 SiO_2_, 1.0 B_2_O_3_, 22.0 CaO, 6.0 Na_2_O, 2.0 P_2_O_5_, 11.0 K_2_O, and 5.0 MgO). They utilized their data to generate a time–temperature–transformation (T–T–T) curve for both bioactive glasses, enabling a comparative analysis between them. The authors reported that 13-93 has a T_g_ of 612 ± 5 °C and 1-98 has a T_g_ of 608 ± 5 °C, in agreement with Fu [77,78]. According to the DTA data, the crystallization peaks of 13-93 and 1-98 occur at 1038 ± 6 °C and 958 ± 6 °C respectively; on the other hand, the crystallization region is much wider than other glasses. In fact, T_c,onset_ is reported to be around 700 °C for both glasses, and the exothermic region is much wider than 45S5. According to the authors’ findings, thermal treatment at temperatures exceeding 800 °C resulted in the predominant crystallization of wollastonite (CaSiO_3_) in both glass compositions, as confirmed by X-ray diffraction analysis. The crystallites were observed to be needle-shaped, with dimensions ranging between 20 and 40 µm [78]. The presence of a wide crystallization region observed in the DTA data of the 13-93 and 1-98 BGs indicates their potential as promising candidates for biomedical applications. Thanks to their thermal behavior, these glasses could provide greater control over crystallization, which can be critical for tailoring their properties to suit specific biomedical needs.

More recent investigations include the development of a novel bioactive glass named BGMS10 (composition in mol.%: 47.2 SiO_2_, 25.6 CaO, 2.3 Na_2_O, 2.6 P_2_O_5_, 2.3 K_2_O, 10.0 MgO, and 10.0 SrO), with an even wider sintering window of about 210 °C [79]. The authors studied the thermal behavior of the BG and observed that the crystallization of CaSiO_3_ takes place within the BG structure at temperatures exceeding 880 °C. This indicates a considerably broad sintering window, as the T_g_ and T_c,onset_ were reported to be 670 and 880 °C, respectively. The research indicates that these favorable thermal properties are accompanied by equally promising biological properties [79,80,81]. Even though more work is needed in order to optimize this BG, biocompatibility tests have demonstrated that BGMS10 exhibits favorable interactions with living tissue, making it a viable solution for various applications [82].

A recent study led to another bioactive glass, named Bio_MS, with composition (in mol.%) 46.1 SiO_2_, 31.3 CaO, 5.0 Na_2_O, 2.6 P_2_O_5_, 5.0 MgO, and 10.0 SrO. Bio_MS has a T_g_ of about 638 °C, and the differential thermal analysis revealed a crystallization peak at 859 °C. This glass exhibits a reduced tendency to crystallize, coupled with promising sintering properties. Furthermore, extensive cytotoxicity assessments have been performed, demonstrating its biocompatibility. Most notably, Bio_MS displays excellent bioactivity and facilitates bone differentiation, making it highly suitable for biomedical applications [83].

ICIE16 is an enhanced biocompatible glass composition, produced using the sol–gel method, specifically formulated to enhance the workability of BGs. Its composition consists of 48.0 wt.% SiO_2_, 6.6 wt.% Na_2_O, 32.9 wt.% CaO, 2.5 wt.% P_2_O_5_, and 10.0 wt.% K_2_O. Westhouser et al. conducted a study in which they successfully thermally treated and sintered ICIE16 at 690 °C, resulting in the production of a sintered amorphous material. This is in contrast to the behavior of 45S5 glass composition [84]. Wu et al. studied the crystalline phases obtained from the heat treatment of ICIE16 BG scaffolds. They reported that the crystallization of two main phases (K_3_Na(SO_4_)_2_ and Na_2_CaSi_3_O_8_) takes place at temperatures exceeding 700 °C [85]. In contrast, Nommeots-Nomm et al. identified Ca_2_Si_4_Ca_3_(PO_4_)_2_ as the main crystalline phase [86]. This discrepancy is likely attributed to several factors, such as variations in heat treatment protocols and slight differences in the glass composition itself, which may induce significant reactions during the treatment process. However, ICIE16 is reported to be a potential candidate for use in sintered manufacts thanks to its relatively low T_g_ and high viscous flow. Given the complexity of the subject, further investigation is necessary to comprehensively identify all the factors contributing to the sinter-crystallization of bioactive glasses.

Table 1 provides a summary of the glass transition and crystallization temperatures for the main BG compositions discussed above.

## 5. Ion Substitution to Improve Thermal Properties of BGs

To tackle the various challenges typically associated with thermal treatments of BGs, scientists have delved into the incorporation of ions into the glass composition to customize the thermal properties of BGs according to specific requirements. In this section, the main effects of different ions on the thermal properties of BGs are discussed. The main objective is to examine the reported influence of ions on their respective BGs and explore the potential advantages of these modifications in enhancing the thermal properties of BGs for biomedical applications.

The mixed alkali effect (MAE) refers to the phenomenon where the inclusion of two or more alkali metal ions (such as Na, K, and Li) in a glass composition can lead to an enhancement of the properties of BGs, such as improved thermal stability, electrical conductivity, and mechanical strength [14]. The effect is believed to arise from changes in the glass structure, as a result of the presence of multiple alkali cations and the difference in the atomic sizes and bonding of the ions [87]. The MAE has been extensively studied in various BG systems and has shown to be promising for enhancing the properties of BGs for biomedical applications. Mixing different alkali ions in the glass structure has been shown to reduce the tendency of the glass to crystallize by introducing distortions in the structure of the glass. This facilitates the preservation of the material’s amorphous structure. All of these factors contribute to enhancing the viscous flow properties of the glass, thereby facilitating improved sintering of the BG [14,88].

Specifically, Moghanian et al. conducted a study on the use of lithium as an additive ion in 58S BG, a bioactive sol–gel-derived glass. The authors found that lithium has a tendency to broaden the endothermic crystallization peak while lowering the crystallization peak temperature from 980 to 952 °C [89]. Maçon et al. studied the interaction between lithium and silica in a Si–Li sol–gel bioactive glass system. They discovered that lithium could act as nucleation site for Li_2_SiO_2_ phase [90]. This is probably due to the fact that the lithium ion is the smallest among the alkali metals, and its presence alone in the composition does not favor the broadening of the sintering window. However, it does lower the T_g_ of the BG. Conversely, potassium, being larger than sodium and lithium, introduces distortions in the glass lattice, thereby impeding crystallization [91,92].

Furthermore, the substitution of K ions in 45S5 for Na ions can slightly improve the mechanical properties of the material. This can be ascribed to the lattice distortion effect caused by potassium, resulting in improvements in microhardness, fracture toughness, and Young’s modulus [92]. Moreover, the research has shown that increasing the total calcium content and substituting potassium for sodium may lead to improved thermal properties. However, it should be noted that this modification also results in a stronger glass network, which may have the unintended effect of slowing down the deposition of hydroxyapatite in simulated body fluid (SBF), as well as in vivo [93].

Strontium has been extensively studied as a substitute ion for calcium in various BG compositions. Generally, strontium acts as a network modifier, causing the expansion of the glass network. As strontium is a larger ion than calcium, this leads to a weakening of the lattice structure. Consequently, as strontium substitution increases, there is a linear decrease in the glass transition temperature. Furthermore, Sr substitution for Ca causes an increase in the density of the glass [94,95]. Fujikura et al. reported that, with increasing strontium substitution in a 45S5-based glass, there was a decrease in crystallization temperature [95]. XRD analysis showed the crystallization of combeite phase during lower-temperature heat treatment. At temperatures exceeding 820 °C, the authors reported the crystallization of strontium-substituted combeite (Na_2_Sr_2_Si_3_O_9_) and a ring-structured silicate phase (Sr_3_(Si_3_O_9_)) [95]. On the other hand, Massera et al. reported a different trend in a S53P4-based composition [94]. They found that the crystallization peak temperature decreases up to 5 mol.% SrO in the glass composition, but then increases for higher concentrations. However, the authors specified that the combination of these two effects did not lead to a significant widening of the sintering window [94].

Bellucci et al. investigated the presence of strontium and magnesium in BGMS10 and its “parent” glass BG_Ca_Mix [79]. They reported that magnesium has a similar effect on the bioactive glass as strontium, particularly concerning the thermal stability of the glass. However, the presence of Sr and Mg has extensively been reported to have positive effects on bone tissue formation [79,96]. Moreover, magnesium has been shown to lower the glass transition temperature in BGs belonging to the CaO–MgO–P_2_O_5_–SiO_2_ system. This is attributed to the weaker Si–O–Mg bond compared to Si–O–Si, resulting in a less robust network structure. This effect is paired with the ability of magnesium to inhibit crystallization of both the BG and the apatite layer deposited after SBF immersion [97]. Evidence suggests that magnesium is incorporated in the apatite-like crystals, decreasing the number of calcium–phosphorus nucleation sites, and resulting in the stabilization of the amorphous structure [98,99].

Zinc has been incorporated in both sol–gel and melt–quench (45S5) BG, resulting in a slight decrease in crystallization and nucleation temperatures. Similarly, substituting zinc for magnesium in BGMS10 also leads to a greater decrease in glass transition and crystallization temperatures. [100]. For instance, the addition of 2 mol.% zinc to the glass composition in place of magnesium can result in a drop in T_g_ from 670 to 622 °C and in T_c_ from 932 to 881 °C [100]. XRD analysis performed by Srivastava et al. revealed that the presence of zinc ions did not affect the crystalline phases, which remained Na_2_Ca_2_Si_3_O_9_ and Na_2_CaSi_3_O_8_ [101]. In contrast, Shruti et al. [102] observed the formation of a Zn_2_SiO_4_ phase after heat treatment at 700 °C. However, the difference in glass composition heavily influenced the crystallization process [101,102]. Nevertheless, the use of zinc ions in glass compositions has some significant drawbacks due to its biological effects. Concentrations of zinc ions over 1 mol.% have been found to slow the formation of apatite, which is undesirable for biomedical applications [101,102]. Additionally, there is a potential risk of cytotoxicity associated with higher concentrations of zinc ions in the glass [100]. Wetzel et al. conducted an investigation into the substitution of small amounts of magnesium or zinc for calcium in the 45S5 bioactive glass, ranging from 2.5 to 15 mol.% [103]. The results showed that the incorporation of these ions improved the processability of the glass. Due to the higher field strength of Mg^2+^ and Zn^2+^ compared to Ca^2+^, their substitution led to an increase in crystallization temperature and a decrease in T_g_. The researchers further noted that even a modest substitution of 2.5 mol.% was sufficient to induce this shift, while higher substitutions did not yield significant improvements in the outcomes [103,104]. These seemingly contrasting results may be attributed to different interactions between ions within the glass structure, which give rise to complex and not easily predictable thermal behaviors.

Research has also focused on the incorporation of barium in bioactive glasses. In particular, researchers have investigated the impact of substituting barium oxide for both silicon and calcium. Arepalli et al. studied barium BaO substitution for SiO_2_ in 45S5 [105]. They discovered that barium functions as a modifier of the glass network, leading to a decrease in both the crystallite nucleation temperature and the T_c_ of the glass. Specifically, the nucleation temperature dropped from 614 to 542 °C, while T_c_ decreased from 760 to 680 °C. The XRD analysis demonstrated that the primary crystalline phase was Na_2_Ca_2_Si_3_O_9_. Khoeini et al. opted to substitute barium for calcium in 45S5 [106]. Their findings are analogous to those of Arepalli et al. [105] and indicate that barium functions as a modifier, weakening the glass structure. Interestingly, Khoeini et al. [106] did not observe a reduction in the number of oxygen bonds in the glass structure as reported by Arepalli et al. [105]. Their research led to the conclusion that barium is inserted into the glass structure as an interstitial element [106]. This leads us to infer that, while barium may exhibit promising biological properties for specific applications, it does not seem to enhance the thermal performance of bioactive glass.

Cobalt has been the subject of investigation as a potential substitution ion in BGs in many research studies. Vyas et al. examined the effects of partially substituting cobalt for silicon in 45S5-based glass compositions, varying the cobalt content from 0 to 4 mol.% [107]. The results showed a general improvement in the mechanical properties of the Co-containing glass. Specifically, the Young’s modulus, shear modulus, bending strength, and density exhibited a linear increase with cobalt substitution, up to a maximum of 3 mol.%. It was observed that cobalt acts as an intermediate oxide up to 2 mol.%, after which it acts as a network modifier. This transition also affects the mechanical properties of the glass, which do not increase linearly over this limit [107]. Moreover, cobalt addition seems to lead to a weakening of the glass network, since Si–O–Co bonds are weaker than Si–O–Si. This has an effect on the T_g_ of the material. DTA data showed that glass transition temperature decreases with increasing Co content [107,108]. However, Co substitution also lowers the crystallization peak temperature from 718 °C to 616 °C. The formed crystalline phases were identified through XRD analysis of thermally treated samples, revealing the presence of two major cobalt-free phases: Na_2_Ca_2_Si_3_O_9_ and Na_2_CaSi_3_O_8_ [107,109]. This observation further supports cobalt’s role as an intermediate oxide in the glass composition.

Zirconium is another element that has recently been used for ion substitution in BGs. Kang et al. gradually replaced sodium with increasing fractions (up to 12%) of zirconium oxide in 45S5 [110]. Zirconium was found to act as a network modifier, increasing the number of bridging oxygen bonds and leading to a more rigid glass structure. This led to improved mechanical properties of the glass, including greater flexural and compression strength, as well as increased Vickers hardness [110,111]. Furthermore, the presence of zirconium had an impact on the T_g_ of the BG, with an increase in zirconium content corresponding to a higher glass transition temperature. Unfortunately, zirconia is a well-known inert biomaterial, and the presence of zirconium in the glass structure can inhibit its bioactivity [111].

**Figure 2:**
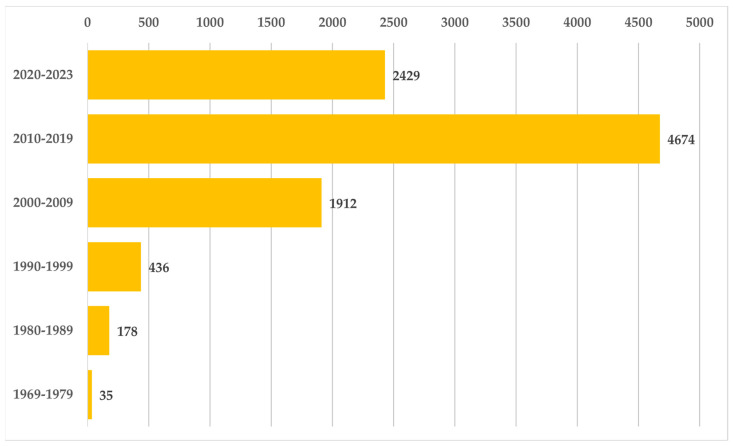
Number of scientific papers on BGs across different decades from the inception in 1969 to 2023. Notably, in the 2020–2023 interval, the publication count is already conspicuous. Data were retrieved via Scopus, with keywords “bioactive glass” or “bioglass”.

The field of BGs has gained unprecedented interest. The number of scientific papers regarding BGs is ever growing, as illustrated in Figure 2. The hope is that this active academic engagement will lead to other interesting discoveries about the effects of different ions on the thermal, mechanical, and biological properties of BGs.

Table 2 reports a summary of the effects of ions on mechanical and thermal properties of BGs discussed in this section.

## 6. Conclusions

In conclusion, BGs show great promise and offer a wide range of potential applications. Some of these applications require heat treatments to enhance the mechanical properties of the glasses or to sinter the BGs powders. However, the crystallization behavior of the “gold” standard 45S5 BG during thermal treatments is still a subject of debate, particularly regarding the main crystalline phase formed after the first crystallization step, whether it is Na_2_Ca_2_Si_3_O_9_ or Na_2_CaSi_2_O_6_.

Despite this challenge, significant progress has been made in improving the thermal properties of these materials through sol–gel synthesis, new glass formulations, and ion substitution. These advancements have opened up new possibilities for the utilization of BGs in areas such as scaffolds, composites, and thermally sprayed coatings. Nonetheless, further research is necessary to fully unlock the potential of these remarkable materials. Continued investigation into the effects of different ions on sintering and crystallization is crucial for further advancements in the field of BGs. Lastly, it is important to investigate the impact of glass composition on thermal residual stresses that occur as a result of the mismatch between the coefficients of thermal expansion of BGs and other phases, such as in composites or BG coatings. This investigation could be conducted through numerical simulations [112] to better understand the role of glass composition in managing thermal stresses.

## Figures and Tables

**Table 1:** Summary of glass transition (T_g_) and crystallization peak (T_c_) and onset (T_c,onset_) temperatures of some relevant BGs.

BG	Glass Transition Temperature T_g_	Crystallization Temperatures: T_c,onset_ (Onset) and T_c_ (Peak)	Ref.
45S5	550 °C	T_c,onset_ = 610 °C	[62]
S53P4	561 °C	T_c_ = 748 °C	[61]
13-93	612 °C	T_c,onset_ = 714 °C, T_c_ = 1038 °C	[77]
1-98	608 °C	T_c,onset_ = 700 °C, T_c_ = 958 °C	[78]
BGMS10	670 °C	T_c,onset_ = 880 °C, T_c_ = 932 °C	[79]
Bio_MS	638 °C	T_c,onset_ = 817 °C, T_c_ = 859 °C	[83]
ICIE16	575 °C	T_c,onset_ = 725 °C	[85]

**Table 2:** Summary of the effect of different ions on mechanical and thermal properties of BGs.

Ion	Parent BG	Substituted Ion	Effect	Refs.
Mixed alkali metals	-	Na	Improved thermal stability	[14,88]
			Improved mechanical properties	
			Hinders crystallization	
			Broader sintering window	
Li	58S	Addition	Broader crystallization peak	[89]
			Lower crystallization temperature (from 980 to 952 °C)	
			New crystallized phase	
K	45S5	Na	Improved mechanical properties	[92]
Sr	S53P4	Ca	Higher density (from 2.66 to 3.03 g/cm^3^)	[94]
			New crystallized phase	
	45S5	Ca	Lower glass transition temperature (from 539 to 497 °C)	[95]
			Lower crystallization temperature (from 665 to 641 °C)	
Mg	-	Ca	Lower glass transition temperature (with increasing Mg content)	[97]
			Inhibit crystallization	
			Less robust network	
	-	Addition	More stable amorphous phase	[99]
Zn	45S5	Ca	Lower glass transition temperature (from DTA plot)	[101,102]
			Lower crystallization temperature (from DTA plot)	
			Improved mechanical properties	
			New crystallized phase (debated)	
	BGMS10	Mg	Lower glass transition temperature (from 670 to 631 °C)	[100]
			Lower crystallization temperature (from 880 to 847 °C)	
Ba	45S5	Si	Lower crystallization onset temperature (from 614 to 542 °C)	[105]
			Lower crystallization temperature (from 760 to 681 °C)	
	45S5	Ca	Lower glass transition temperature (from 652 to 641 °C)	[106]
			Lower crystallization temperature (from 853 to 811 °C)	
			Reduced number of oxygen bonds	
Co	45S5	Si	Improved mechanical properties	[107]
			Lower crystallization onset temperature (from 535 to 455 °C)	
			Lower peak crystallization temperature (from 718 to 616 °C)	
Zr	45S5	Na	Improved mechanical properties	[110]
			Increased number of bridging oxygen bonds	

## Data Availability

Not applicable.
